# Jean-Alexandre Barré (1880–1967)

**DOI:** 10.1007/s00415-017-8673-y

**Published:** 2017-11-15

**Authors:** Michał K. Owecki, Piotr Skalski, Anita Magowska

**Affiliations:** Department of History of Medical Sciences, Poznań University of Medical Sciences, ul. Przybyszewskiego 37A, Poznań, Poland

**Fig. 1:**
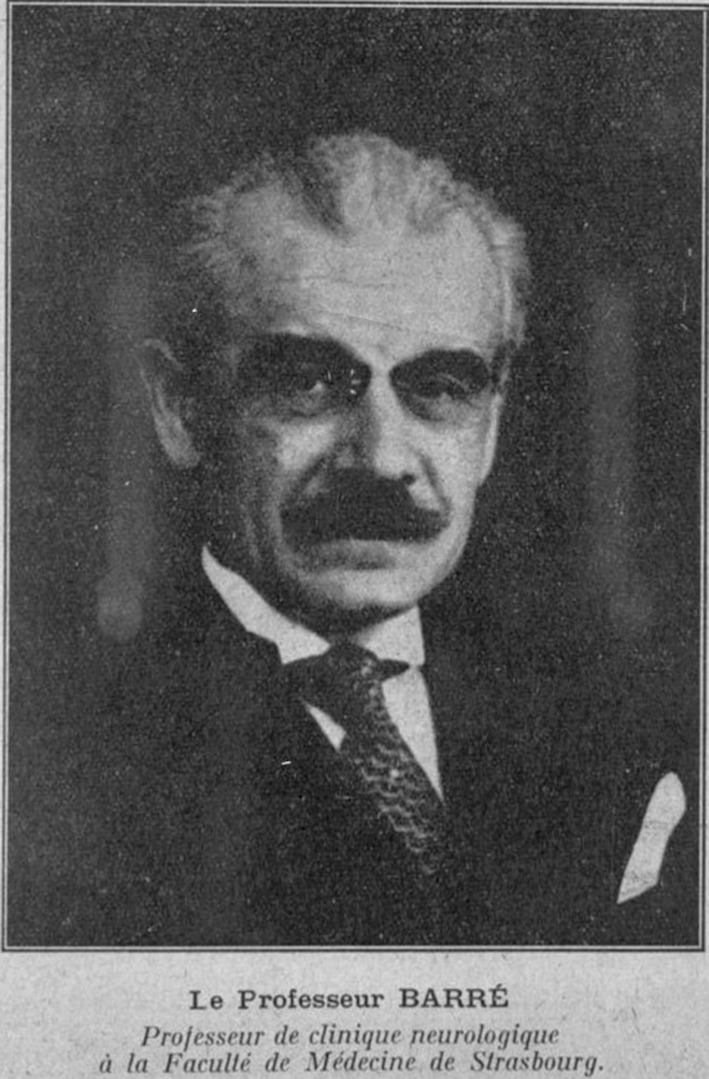
Jean-Alexandre Barré (public domain)

Jean-Alexandre Barré (Fig. 1) was a French neurologist, now mostly known for an early description of acute immune-mediated inflammatory polyneuropathy, a heterogeneous neurological condition classified and grouped under the eponym Guillain-Barré syndrome. A skillful and perceptive clinician, he was also the author of the Barré test, a diagnostic maneuver still sometimes used in everyday neurological practice.

Barré was born on May 25, 1880, in Nantes in France. He studied medicine at the University of Nantes and, following his graduation, began his medical career at a hospital in his hometown as an intern, with the idea of becoming a surgeon. In 1901, he completed his compulsory military service and, 5 years later, moved to Paris, where he successfully applied for an internship. It was during that time that he met Joseph Babiński (1857–1932), a masterful clinician of Polish origin, who introduced Barré to the fascinating world of neurology. Meeting Babiński influenced Barré’s life: he abandoned his surgical plans and began his neurological career. He completed his medical training under the guidance of Dr Alexandre-Achille Souques (1860–1944) and Professor Pierre Marie (1853–1940). Barré’s interests at that time were focused on syphilis and the skeletal changes caused by the disease. In 1912, he obtained his doctorate degree for an original thesis entitled “Osteoarthropathies in spinal syphilis. A critical study and a new conception” [1].

In the following years, Barré continued his neurological work as assistant to Babiński until the outbreak of World War I (1914–1918). The war brought new challenges for the medical staff of the time: millions of soldiers suffered severe psychological and somatic injuries of new types and on a scale never experienced before. Barré began his military service as a member of a front-line ambulance unit, but was later directed, fortunately, to hospital work within a neurological unit of the Sixth Army in the northern region of France. This relocation not only radically improved his chances of surviving the war, but also gave rise to a successful collaboration with Georges Guillain (1876–1961), the chief physician at the Sixth Army’s neurology center at the time. In 1917, Barré was assigned to the military neurological center in Nantes and afterwards, in 1918, with the end of the war, he gained a position as Director of the Neuropsychiatric Military Center in the Eastern Region of France.

Throughout the war years, Barré effectively combined his military duties and care of injured soldiers with his scientific interests: hundreds of wounded men were not only the subject of his medical attention, but also the inspiration for in-depth clinical analysis. In cases of suspected hysteria as a way of avoiding army service, Barré warned against hastily attributing the symptoms observed to psychological causes and recommended a careful neurological examination of each patient. In close collaboration with Guillain, he studied the clinical presentation and course of numerous cases of peripheral and central nervous system traumatic lesions. The two men’s research work on wounded soldiers resulted in improved neurological examination techniques and contributed new insights to localizing symptomatology, in particular of spinal cord injuries. Their observations supported the conception of the early surgical management of spinal cord trauma and accompanying vertebral fractures. However, a high mortality rate due to bacterial infections remained an impassable obstacle for Barré and his colleagues in that pre-antibiotic era [2].

Neurology owes the early reporting of acute inflammatory demyelinating polyradiculoneuropathy to Barré and Guillain, the condition today referred to by an eponym combining the two co-workers: Guillain–Barré syndrome. The first clinical descriptions of the disease had come from earlier authors, such as Auguste François Chomel (1788–1858), who reported an epidemic incidence of the syndrome in Paris in 1828 [3], and Jean Landry (1826–1865), who, in 1859, documented ten cases using the term “acute ascending paralysis” [4]. However, the report by Barré and Guillain was the first supported by both cerebrospinal fluid analysis and the newly emerging techniques of electrophysiological studies. In 1916, Guillain and Barré turned their attention to two similar cases of young soldiers, both of whom had developed partial flaccid paralysis accompanied by areflexia; the weakness would spontaneously recover with time. Biochemical examination of cerebrospinal fluid samples revealed increased levels of albumin, but without a cytological response. The description of the syndrome was prepared in close collaboration with André Strohl (1887–1977), a doctor of both physics and medicine, who performed electrophysiological tests on the soldiers. In the same year, all the authors published their findings in a French medical journal [5]. Only 4 years later, in a book broadly presenting their neurological experiences from the war, Barré and Guillain again presented the case studies, but did not mention Strohl as co-author [2]. When, in 1927, Draganesco and Claudian used the term “Guillain–Barré syndrome” for the first time in the medical literature [6], Barré and Guillain neither objected nor complemented the newly established eponym with Strohl’s name [7]. Thus, despite his contribution, André Strohl was eliminated from the eponymic designation of the syndrome.

With the end of World War I, Barré was appointed Professor of Neurology at Strasbourg, at the age of 39. He focused his interests on vestibular functions and pathology and, in 1927, founded a French-language journal dedicated to the common problems of neurology, ophthalmology and otology: the *Revue d’oto*-*neuro*-*ophtalmologie*.

Barré proved his clinical acumen by the test he established to detect slight pyramidal paresis. In 1919, he described an examination technique for suspected lower-limb weakness: as a patient in a prone position tries to maintain both legs separately at a 90° angle from the bed, the extremity on the paretic side will lower. Barré warned against false positive interpretations of the test in the case of the painful restriction of leg joint mobility [8]. In 1920, he presented an analogous test, dedicated to identifying pyramidal weakness of the upper extremities: the arms outstretched, the palms facing each other and the fingers spread [9].

In a large review article in 1937, Barré proposed a novel variant of the leg drift test, to be carried out in a supine position with the lower extremities flexed 90° at the hips and knees; this was actually a modification of the Giovanni Mingazzini (1859–1929) leg maneuver, described as early as 1913. Similarly, in the same paper, Barré presented the arm drift test in the way also recommended previously by Mingazzini. However, Barré noticed that in pyramidal syndromes, a paretic arm not only gradually lowered, but also lost its initial extended position and became slightly flexed at the joints [10]. Thus, the test still performed today in neurological practice, which was originated by Mingazzini but then adopted and broadly described by Barré, has generally been referred to as the Barré test.

Barré dedicated himself to neurology but he also had another passion—music. He loved classical music; his first wife, who died young, was a pianist, and his daughters were educated in music.

In 1953, on his return from a congress in Lisbon, Barré suffered a stroke, which resulted in hemiparesis. In spite of the condition, Barré battled his disability and continued to participate in medical scientific meetings. Jean-Alexandre Barré passed away on April 26, 1967, in Strasbourg.
